# Harnessing adiponectin for sepsis: current knowledge, clinical insights and future therapies

**DOI:** 10.1186/s13054-025-05516-2

**Published:** 2025-07-12

**Authors:** Stefano Gianoli, Justin Tang, Kirsten C. Odegard, Koichi Yuki, Sophia Koutsogiannaki

**Affiliations:** 1 Department of Anesthesiology, Critical Care and Pain Medicine, Cardiac Anesthesia Division, Boston Childrens Hospital, 300 Longwood Ave, Boston, MA 02115 USA; 2 Department of Anaesthesia, Harvard Medical School, Boston, MA 02115 USA; 3 Department of Immunology, Harvard Medical School, Boston, MA 02115 USA; 4 Broad Institute of Harvard and MIT, 415 Main St, Cambridge, MA 02142 USA; 5 Department of Biomedical Science, University of Guelph, Guelph, ON N1G 2W1 Canada

**Keywords:** sepsis; adiponectin; immune system; therapeutic interventions

## Abstract

Adiponectin, a key adipokine primarily secreted by adipocytes, plays crucial roles in metabolic homeostasis and inflammation, exhibiting anti-diabetic, anti-atherogenic, and anti-inflammatory properties. Its various isoforms and signaling via receptors like AdipoR1, AdipoR2, and T-cadherin contribute to its diverse biological functions. Sepsis is a life-threatening syndrome triggered by a dysregulated host response to infection, leading to systemic inflammation, multi-organ failure, and high mortality, currently lacking specific treatments. Preclinical studies largely suggest a protective role for adiponectin, demonstrating that its deficiency exacerbates inflammation and endothelial dysfunction, while its administration or agonism improves outcomes in experimental sepsis models. Clinical findings, however, present a complex picture, with inconsistent correlations between adiponectin levels and sepsis outcomes reported, suggesting its potential as a dynamic biomarker influenced by disease stage, patient heterogeneity, and isoforms, rather than a simple prognostic factor. Notably, glucagon-like peptide-1 receptor agonists (GLP-1RAs), used in obesity and diabetes management, have been shown to increase adiponectin levels, linking metabolic therapies to potential sepsis immunomodulation. Consequently, targeting adiponectin signaling, either directly with adiponectin mimics like AdipoRon or indirectly via strategies like GLP-1RA administration, represents a promising therapeutic approach for sepsis. Harnessing the adiponectin axis holds potential for advancing precision medicine in critical care, necessitating further research into adiponectin-based interventions and synergistic metabolic therapies to improve sepsis outcomes.

**Graphical abstract:**

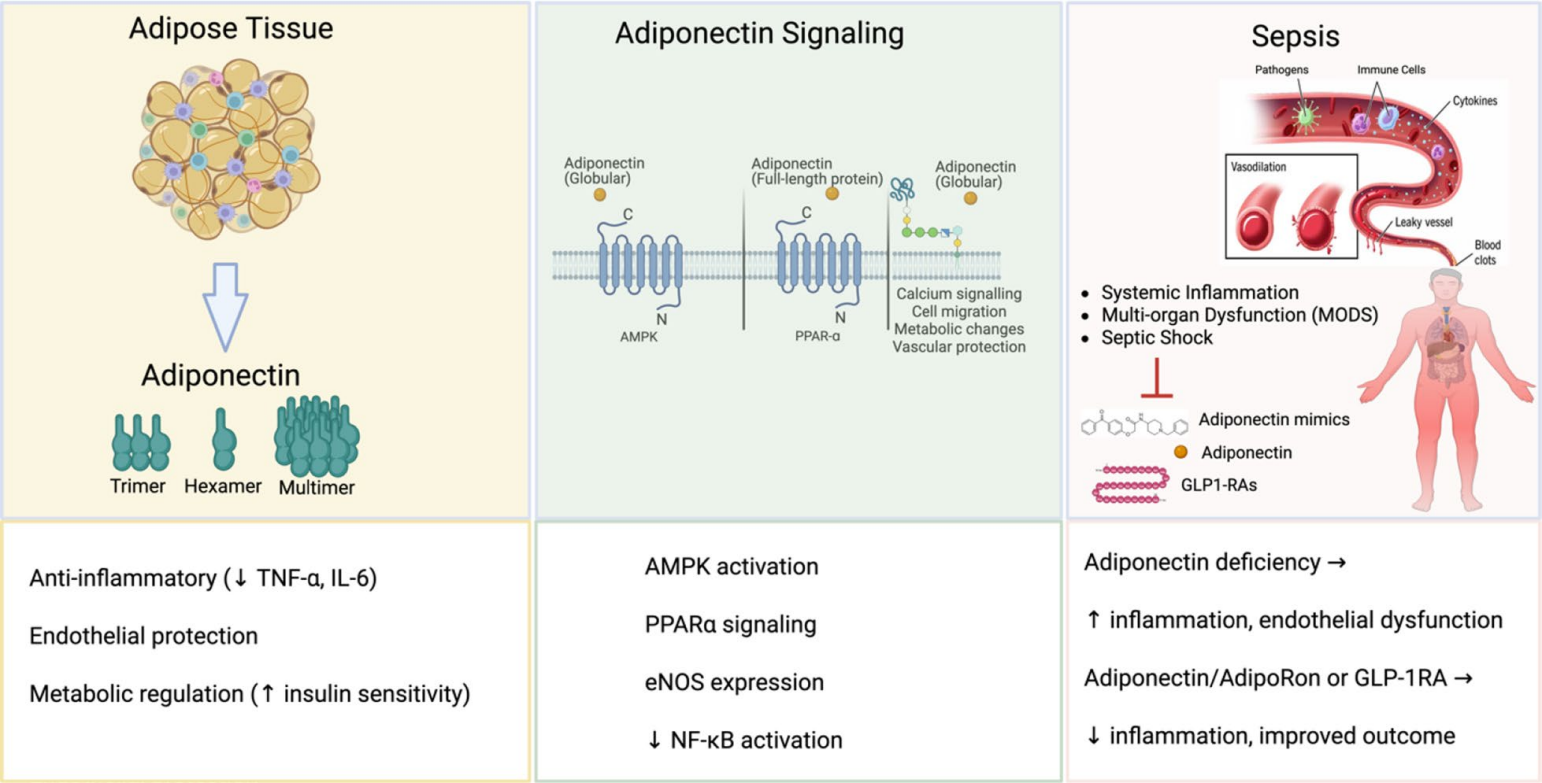

## Introduction

Adiponectin is a highly abundant adipokine primarily secreted by adipocytes in adipose tissue (also known as"fat") and is often termed the"guardian angel adipokine"due to its multifaceted biological functions [1]. It plays a pivotal role in maintaining metabolic and inflammatory homeostasis through its well-documented anti-diabetic, anti-atherogenic, anti-inflammatory, and anti-cancer properties [2, 3]. Notably, adiponectin levels inversely correlate with body mass index (BMI) and visceral adiposity, linking it to obesity-related conditions such as metabolic syndrome, type 2 diabetes, insulin resistance, and cardiovascular diseases, including hypertension [4–10]. Adiponectin exists in various isoforms—low, medium, and high molecular weight (LMW, MMW, HMW)—which exert their effects via receptors AdipoR1, AdipoR2, and T-cadherin, differentially expressed across tissues and immune cells [11–13]. These distinct isoforms and receptor interactions underscore the complexity of adiponectin signaling and its broad spectrum of physiological effects [14]. Given its extensive regulatory role, adiponectin has been recognized as a potential therapeutic target in metabolic and inflammatory diseases. While adiponectin’s function in chronic diseases associated with metabolic dysfunction is well established, its role in acute conditions such as sepsis remains less clearly defined. Understanding adiponectin's impact on immune modulation and inflammatory responses in sepsis may provide novel insights into its potential as a biomarker or therapeutic target in critical illness.

Sepsis is a life-threatening syndrome caused by a dysregulated host response to infection, leading to systemic inflammation, tissue damage, multi-organ failure, and high mortality [15, 16]. According to the World Health Organization (WHO), sepsis affects approximately 48.9 million people globally each year, contributing to 11 million deaths—nearly 20% of all global fatalities [17]. It is primarily triggered by bacterial infections but can also arise from viral and fungal pathogens, as well as post-surgical complications, particularly in nosocomial settings [18, 19]. Prompt recognition and treatment are critical to improving outcomes, as sepsis can progress rapidly to septic shock and multiple organ failure. Despite advances in critical care, sepsis mortality remains high, and no specific targeted therapy exists. Current management relies on early diagnosis, source control, antibiotics, and supportive care [20, 21]. While historically viewed as progressing from an initial hyperinflammatory phase to subsequent immunosuppression, current evidence shows that pro- and anti-inflammatory processes frequently co-exist from early stages [22]. This concurrent immune activation contributes to the complexity of sepsis and underscores the need for more nuanced, patient-tailored therapeutic approaches.

Towards this therapeutic goal, identifying sepsis mediators along with risk factors such as weakened immune systems, chronic illnesses, advanced age, or the presence of invasive devices is vital in preventing sepsis and ensuring early detection in high-risk populations [23–26]. Amidst the hundreds of mediators implicated in sepsis, adiponectin is uniquely positioned due to its dual role as a metabolic hormone and immunomodulator. As discussed extensively in this review, beyond its well-characterized functions in glucose and lipid homeostasis, adiponectin can exert both pro-and anti-inflammatory responses in sepsis, along with endothelial-protective, and organ-preserving effects that are increasingly recognized in both experimental and clinical models of sepsis. Fluctuations in circulating adiponectin levels correlate with sepsis severity and outcomes, suggesting a potential role as both a biomarker and a therapeutic target [27–31]. In addition, obesity has been identified as a significant risk factor for sepsis and emerging evidence suggests a complex interplay between adiponectin, obesity, and sepsis outcomes [32–34]. Thus, modulating adiponectin signaling or mimicking its effects has emerged as a potential therapeutic strategy in sepsis. Moreover, recent advances in metabolic therapy have brought renewed attention to pathways that intersect with adiponectin signaling. Notably, glucagon-like peptide-1 receptor agonists (GLP-1RAs), including widely used agents such as Ozempic, have demonstrated the ability to elevate adiponectin levels while improving metabolic and inflammatory profiles in patients with obesity and diabetes [35, 36]. These agents, which have gained substantial clinical relevance and have been recently suggested for sepsis treatment [37], further highlight the therapeutic potential of targeting adiponectin-related pathways in sepsis. Taken together, adiponectin represents a compelling focus for translational research in sepsis, offering mechanistic insight into the metabolic-immune interface and potential pathways for precision therapies. This review synthesizes pre-clinical and clinical evidence on adiponectin's role in sepsis, examines the complex interplay with obesity, evaluates potential therapeutic strategies targeting adiponectin pathways including adiponectin mimics and GLP-1 receptor agonists, and outlines key future research directions.

### Adiponectin biology

Adiponectin is present at high concentrations in plasma (3–30 μg/ml), which accounts for up to 0.05% of total serum protein. It is a 244 amino acid protein primarily secreted from the adipose tissue and is the most abundant protein secreted by it [38]. Recent studies have shown that adiponectin is also produced by the skeletal muscle cells [39]. In addition, endothelial cells have also been reported to express adiponectin [40]. Adiponectin directly acts on the liver, skeletal muscle, and vasculature through insulin sensitization and anti-inflammatory/anti-atherogenic effects [3, 41]. Since discovering adiponectin in the 1990 s, it has become a widely accepted biomarker for obesity-related diseases such as metabolic syndrome, Type 2 Diabetes mellitus, and atherosclerotic cardiovascular disease and its anti-inflammatory properties are well-documented as well [42–45]. Originally identified almost simultaneously by four independent research groups, adiponectin has been assigned multiple names, including Acrp30, apM1, GBP28, and AdipoQ [46–49]. It mediates its biological effects through three known receptors—AdipoR1, AdipoR2, and T-cadherin—which are distributed across multiple tissues [11, 12] and immune cells [13]. The protein is encoded by the AdipoQ gene on chromosome locus 3q27. The adiponectin protein contains an NH2-terminal hyper-variable region, a collagenous domain of 22 Gly-XY repeats, and a COOH-terminal C1q-like globular domain. The C-terminal globular domain of adiponectin is highly similar to the structure of tumor necrosis factor-α (TNF-α) as well [50].

Adiponectin is synthesized as a 30 kDa monomer that undergoes post-translational modifications and oligomerization, forming distinct isoforms: low (67 kDa LMW), medium (136 kDa; MMW), and heavy (300 kDa; HMW) molecular weight adiponectin [14, 50–52]. The HMW isoform is considered the most biologically active, particularly in its insulin-sensitizing and anti-inflammatory roles [53]. Also, the globular domain of adiponectin (∼18–25 kDa), which can be produced via the action of neutrophil elastase, has been shown to similarly present significant metabolic effects in various tissues [54].

### Adiponectin and sepsis: pre-clinical observations

#### The anti-inflammatory role of adiponectin in experimental sepsis

Experimental research in sepsis models supports that adiponectin plays a critical role in modulating inflammation and vascular integrity during sepsis. Its deficiency has been shown to exacerbate leukocyte and platelet adhesion in the cerebral microcirculation of septic mice, leading to increased blood–brain barrier dysfunction and elevated E-selectin expression. This heightened inflammatory response contributes to the severity of sepsis-associated complications. Notably, blocking E-selectin mitigates these effects, underscoring the role of adiponectin in regulating microvascular inflammation and protecting the integrity of the blood–brain barrier [55]. In support, another study in septic mice also highlights the crucial role of adiponectin in modulating endothelial activation and survival in sepsis. Using both loss-of-function (adiponectin-deficient mice) and gain-of-function (recombinant adiponectin administration) approaches in cecal ligation perforation (CLP) and thioglycolate-induced peritonitis models, researchers found that AdipoQ^−/−^ mice exhibited significantly reduced survival and heightened inflammatory responses. These mice showed excessive neutrophil infiltration, increased levels of key cytokines, including IL-12p70, TNF-α, Monocyte Chemoattractant Protein-1 (MCP-1), and IL-6, and upregulated endothelial adhesion molecules (VCAM-1 and ICAM-1). Recombinant adiponectin administration effectively mitigated these effects, with higher-order adiponectin oligomers playing a central protective role. These findings underscore adiponectin’s potential as a key modulator of endothelial inflammation and survival in sepsis, suggesting a mechanistic link between adiposity and sepsis outcomes [29]. In the context of sepsis-associated encephalopathy (SAE), a severe neurological complication of sepsis, the downregulation of adiponectin receptor 1 (AdipoR1) in the hippocampus is linked to cognitive impairment, synaptic damage, and neuronal loss [56]. Treatment with AdipoRon, a small-molecule agonist of AdipoR1, currently in pre-clinical development for many different metabolic diseases [57], restores the activation of adenosine monophosphate-activated protein kinase (AMPK), reducing neuroinflammation, protecting synaptic integrity, and improving memory function [56]. Beyond neurological effects, adiponectin deficiency has been directly associated with increased mortality in polymicrobial sepsis. Adiponectin-knockout mice exhibited significantly higher mortality rates and elevated levels of inflammatory cytokines such as TNF-α and IL-6. Interestingly, treatment with rosiglitazone, a Peroxisome proliferator-activated receptor gamma (PPAR-γ) agonist that increases adiponectin levels, improved survival in wild-type mice but failed to do so in adiponectin-deficient mice, reinforcing the crucial role of adiponectin in modulating the immune response and enhancing survival during sepsis [30]. A commentary on the above-mentioned study, suggested that the regulation of adiponectin in sepsis may additionally involve the heme oxygenase-1 (HO-1) pathway and its byproduct, carbon monoxide (CO) [58], since studies indicate that CO-releasing molecules can suppress inflammatory cytokine production, modulate metabolism, and influence mitochondrial function, potentially forming an HO-1–adiponectin regulatory axis [59–63]. If validated, this mechanism could open new avenues for therapeutic interventions targeting both inflammation and metabolic dysfunction in sepsis. The impact of adiponectin is particularly relevant in the context of obesity, which alters immune responses and sepsis outcomes. In diet-induced obese mice with early sepsis, microvascular inflammation, characterized by increased leukocyte and platelet adhesion, was more severe than in non-obese controls. However, adiponectin replacement therapy effectively reduced these inflammatory interactions. Mechanistic studies revealed that adiponectin attenuates inflammatory responses in macrophages through both Sirtuin 1 (SIRT1)-dependent and independent pathways, suggesting a broader regulatory role in immune function during sepsis [26]. Further supporting the protective role of adiponectin, studies have demonstrated that its administration significantly reduces mortality in septic mice while suppressing levels of IL-6, TNF-α, and high-mobility group box 1 (HMGB1), a key mediator of inflammation and organ damage in sepsis. A strong correlation between serum and lung HMGB1 expression suggests that adiponectin exerts its protective effects in part by inhibiting HMGB1 signaling [64]. These findings reinforce adiponectin’s potential as a critical modulator of the inflammatory response and a promising target for future sepsis therapies. Table 1 provides a detailed summary of preclinical studies investigating the role of adiponectin in sepsis, supporting either its anti-inflammatory or pro-inflammatory effects.

**Table 1:** Summary of preclinical studies examining the role of adiponectin in sepsis. The table highlights findings that support both anti-inflammatory and pro-inflammatory effects

References	Adiponectin’s effect	Experimental approach	Key mechanism
Anti-inflammatory effects of adiponectin in pre-clinical sepsis			
Teoh et al. [29]	Protects from sepsis mortality	AdipoQ^−/−^ mouse CLP model	eNOS activation via AMPK
Uji et al. [30]	Protects from sepsis mortality	AdipoQ^−/−^ mouse CLP model	Plasma endotoxin, TNF-α, ΙL-6
Vachharajani et al., (2012) [55]	Protects blood–brain barrier	AdipoQ^−/−^ mice + Lipopolysaccharide (LPS)	RhoA/ROCK pathway
Bai et al., (2024) [56]	Protects cognitive function	Septic mice	BDNF upregulation
Hoetzel et al., (2007) [59]	CO enhances adiponectin effects	LPS-treated macrophages	p38 MAPK inhibition
Sawle et al., (2005) [60]	CO-RMs synergize with adiponectin	RAW264.7 cells	Nrf2 activation
Sun et al., (2008) [61]	Prevents endothelial activation	HUVEC + LPS	SIRT1 dependent
Li et al., (2008) [62]	Improves insulin sensitivity	Obese diabetic mice	HO-1 induction
Lancel et al., (2009) [63]	Improves mitochondrial function	Mouse CLP model	PGC-1α biogenesis
Li et al., (2012) [64]	Reduces sepsis mortality	Mouse CLP model	HMGB1 suppression
Pro-inflammatory effects of adiponectin in pre-clinical sepsis			
Karampela et al., (2019) [31]	HMW adiponectin in obese sepsis	Obese mouse CLP model	ERK/IL-10 dysregulation

#### Additional potential mechanisms of adiponectin-mediated protection in sepsis

Although the research of adiponectin specifically in sepsis is relatively limited, there is a plethora of research studying adiponectin’s anti-inflammatory properties in many different contexts, that provides mechanistic insights that could be relevant to sepsis as well [65]. Zhang et al. [43] have recently provided a detailed review of such adiponectin’s diverse cellular and vascular activities associated with its anti-inflammatory role, which could provide further mechanistic insights on its observed protective role in experimental sepsis. In brief, at the cellular level, research suggests that adiponectin regulates immune responses by modulating macrophages, neutrophils, T cells, B cells, dendritic cells, and endothelial cells in different ways [66]. In sepsis, an excessive inflammatory response driven by hyperactivation of innate and adaptive immunity can lead to systemic tissue damage and immune dysregulation. Thus, adiponectin’s effects on modulating immune cells could potentially explain its protective effect in sepsis.

For instance, it has been found that adiponectin inhibits macrophage polarization towards the pro-inflammatory M1 phenotype while promoting the anti-inflammatory M2 phenotype production [67, 68]. Additionally, it suppresses the production of pro-inflammatory cytokines such as TNF-α, IL-6, and IL-12 while enhancing anti-inflammatory IL-10 [69–72]. This could provide an additional mechanism through which adiponectin exerts its anti-inflammatory role in sepsis, since it is known that macrophages play a pivotal role in infection and inflammation, including sepsis, and that in the early stages of sepsis, pro-inflammatory factors encourage macrophages to polarize into M1 phenotype, in an uncontrolled manner that evolves into a cytokine storm, exacerbating inflammation and tissue damage. To suppress excessive inflammation during sepsis, macrophages protect the host from further damage and promote wound healing through polarization into M2 phenotype [73].

In addition, neutrophils that are the most abundant immune cells in the blood, have a central role in sepsis and are main mediators of sepsis-induced organ injury [74]. Under physiological conditions, neutrophils undergo apoptosis to maintain their homeostasis. However, during sepsis, neutrophils experience several functional alterations, including diminished migration capacity, altered antimicrobial activity, and delayed apoptosis, thereby contributing to immune dysfunction and persistent inflammation [75]. Adiponectin has been shown to modulate neutrophil activity by inhibiting chemotaxis, reducing oxidative burst via NADPH oxidase inhibition [76], and decreasing neutrophil apoptosis through AMPK activation and p38 MAPK inhibition [77], which in turn could explain how adiponectin potentially protects tissues and improves survival in sepsis.

Moreover, dendritic cells (DCs) function as antigen-presenting cells, determining T cell responses through cytokine secretion, and adiponectin influences this process by activating AdipoR1 and AdipoR2, leading to increased IL-10 production and reduced NF-κB activation. This results in an overall suppression of antigen-specific T cell responses [78, 79]. By inhibiting the proliferation and activation of pro-inflammatory Th1 and Th17 cells, adiponectin prevents the excessive secretion of IFN-γ and IL-17, which are known to exacerbate sepsis-induced inflammation as well. Additionally, adiponectin enhances regulatory T cell (Treg) function by upregulating FOXP3 and IL-10 expression, promoting immune tolerance which could be beneficial in sepsis through limiting the uncontrolled immune activation that is characteristic of sepsis [80–82]. The suppression of cytotoxic CD8⁺ T cells by adiponectin further reduces inflammatory macrophage activation, curbing tissue damage that is another hallmark in sepsis. In B cells, adiponectin inhibits lymphopoiesis in the bone marrow while promoting differentiation into anti-inflammatory plasma cells via the PI3K/Akt1/STAT3 pathway, which helps shift the immune response toward resolution [83]. Collectively, in the context of sepsis, these effects could contribute to a balanced immune response, reducing excessive inflammation while preserving immune function necessary for pathogen clearance.

**Fig. 1:**
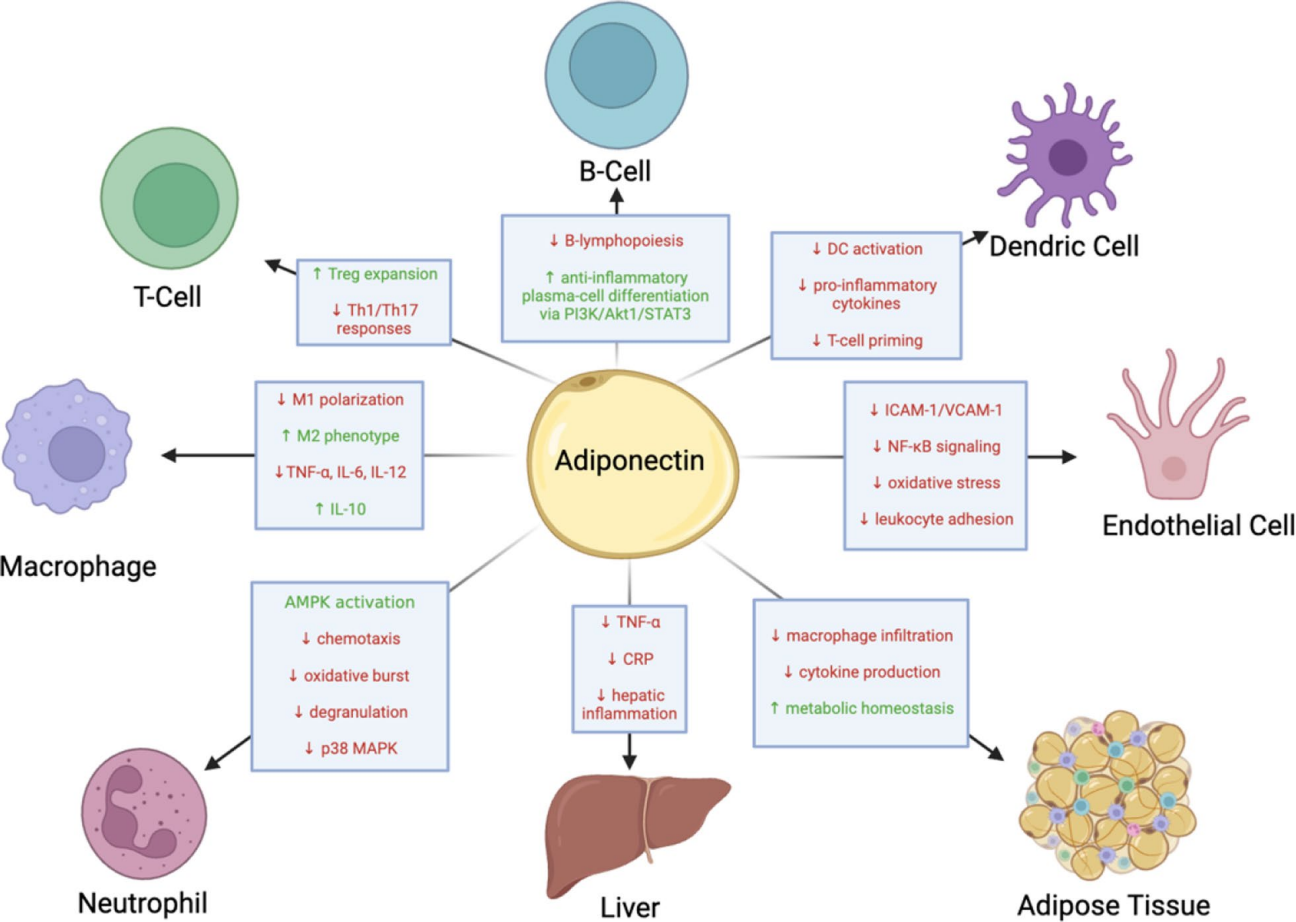
Adiponectin effects on immune cells and tissues. Adiponectin exerts broad immunomodulatory effects, primarily promoting an anti-inflammatory environment. It reduces neutrophil mobility and activation, supports an M2 macrophage phenotype, and enhances T regulatory expansion while suppressing Th1/Th17 responses and dendritic cell activation, leading to lower pro-inflammatory cytokine production. In B cells, adiponectin inhibits lymphopoiesis but promotes differentiation into anti-inflammatory plasma cells via the PI3K/Akt1/STAT3 pathway. It also reduces leukocyte adhesion to endothelial cells by downregulating the ICAM-1/VCAM-1 axis. Additionally, adiponectin mitigates TNF-driven inflammation in the liver and limits macrophage infiltration in adipose tissue. Overall, its predominant effect is immunosuppressive

Beyond immune regulation, adiponectin exerts protective vascular effects by decreasing endothelial expression of adhesion molecules such as ICAM-1 and VCAM-1, thereby limiting leukocyte adhesion and migration into tissues [29, 84], which can provide a beneficial effect against sepsis-mediated tissue injury. It also suppresses Nuclear Factor kappa B (NF-κB) signaling, a central regulator of inflammation, in endothelial cells and macrophages, while reducing oxidative stress to prevent endothelial dysfunction [85–87]. Tissue-specific effects further illustrate adiponectin’s immunoregulatory function: in the liver, it mitigates hepatic inflammation by suppressing TNF-α and C-reactive protein (CRP) production [88–90], while in adipose tissue, it reduces macrophage infiltration and cytokine production, curbing inflammation-driven metabolic disturbances [33, 91]. Taken together, these effects of adiponectin further support its potential anti-inflammatory and protective role in sepsis and sepsis-mediated organ dysfunction (Fig. 1).

#### Adiponectin receptors and their role in sepsis

Adiponectin exerts the above-described effects through three receptors: AdipoR1, AdipoR2, and T-cadherin [92]. While AdipoR1 is primarily expressed in skeletal muscle and innate immune cells, where it activates AMPK to regulate metabolism, AdipoR2 is abundant in the liver and adipose tissue, promoting lipid metabolism through PPARα activation [79]. T-cadherin, is abundantly expressed in endothelial and cardiac tissues and plays a crucial role in adiponectin-mediated cardio-protection [93]. In experimental sepsis models, these receptors play distinct yet complementary roles in regulating immune and metabolic responses to systemic infection. AdipoR1, highly expressed in macrophages and neutrophils, mediates the anti-inflammatory effects of adiponectin by activating AMPK, which inhibits NF-κB signaling and reduces cytokine production [94]. Studies in LPS-induced septic mice have shown that AdipoR1 is downregulated in sepsis-associated encephalopathy (SAE), contributing to neuronal damage and cognitive decline, while activation of AdipoR1 by a receptor agonist (AdipoRon) restores AMPK signaling, reducing neuroinflammation and protecting against SAE-induced brain injury [56].

AdipoR2, predominantly expressed in hepatocytes and adipocytes, plays a metabolic role in sepsis by regulating lipid metabolism and energy homeostasis [12, 13]. During sepsis, metabolic dysfunction leads to increased hepatic lipid accumulation and insulin resistance, exacerbating systemic inflammation. AdipoR2 activation mitigates these effects by promoting PPARα signaling, which enhances fatty acid oxidation and reduces hepatic steatosis [95]. Experimental studies in AdipoR2-knockout mice have demonstrated heightened susceptibility to metabolic disturbances, with impaired glucose utilization and worsened mitochondrial dysfunction [96]. Furthermore, adiponectin-mediated activation of AdipoR2 has been shown to protect against hepatocellular injury by reducing oxidative stress and inflammatory cytokine release [97].

T-cadherin, though lacking a classical signaling domain, serves as a key regulator of adiponectin bioavailability and vascular protection. It is abundantly expressed in endothelial and cardiac tissues, where it facilitates the retention of high-molecular-weight (HMW) adiponectin [11]. Experimental studies suggest that T-cadherin-deficient mice exhibit reduced adiponectin binding to endothelial cells, leading to increased vascular permeability and endothelial dysfunction during sepsis [98]. In contrast, overexpression of T-cadherin enhances adiponectin-mediated endothelial protection, reducing leukocyte adhesion and preserving microvascular integrity. Additionally, T-cadherin has been implicated in cardiac protection, as adiponectin-deficient and T-cadherin-knockout mice display heightened susceptibility to myocardial dysfunction, characterized by reduced ejection fraction and increased cardiac inflammation [93].

**Fig. 2:**
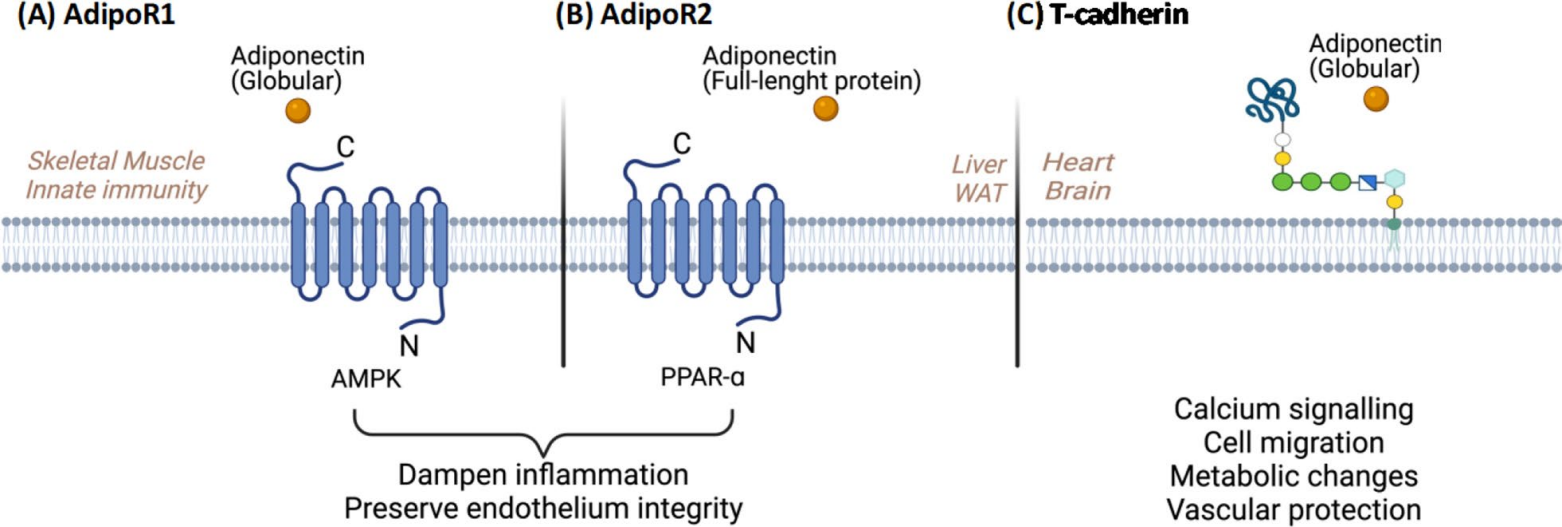
Adipor-1 and Adipor-2 signaling. **(A)** AdipoR1 primarily activates AMPK, which inhibits NF-κB signaling, regulating metabolism, and reducing pro-inflammatory cytokine production. **(B)** AdipoR2 promotes lipid metabolism through PPARα activation. **(C)** T-cadherin lacks a classical signaling domain; however, it is considered a key regulator of Adiponectin bioavailability and vascular protection in the heart, especially during sepsis

**Fig. 3:**
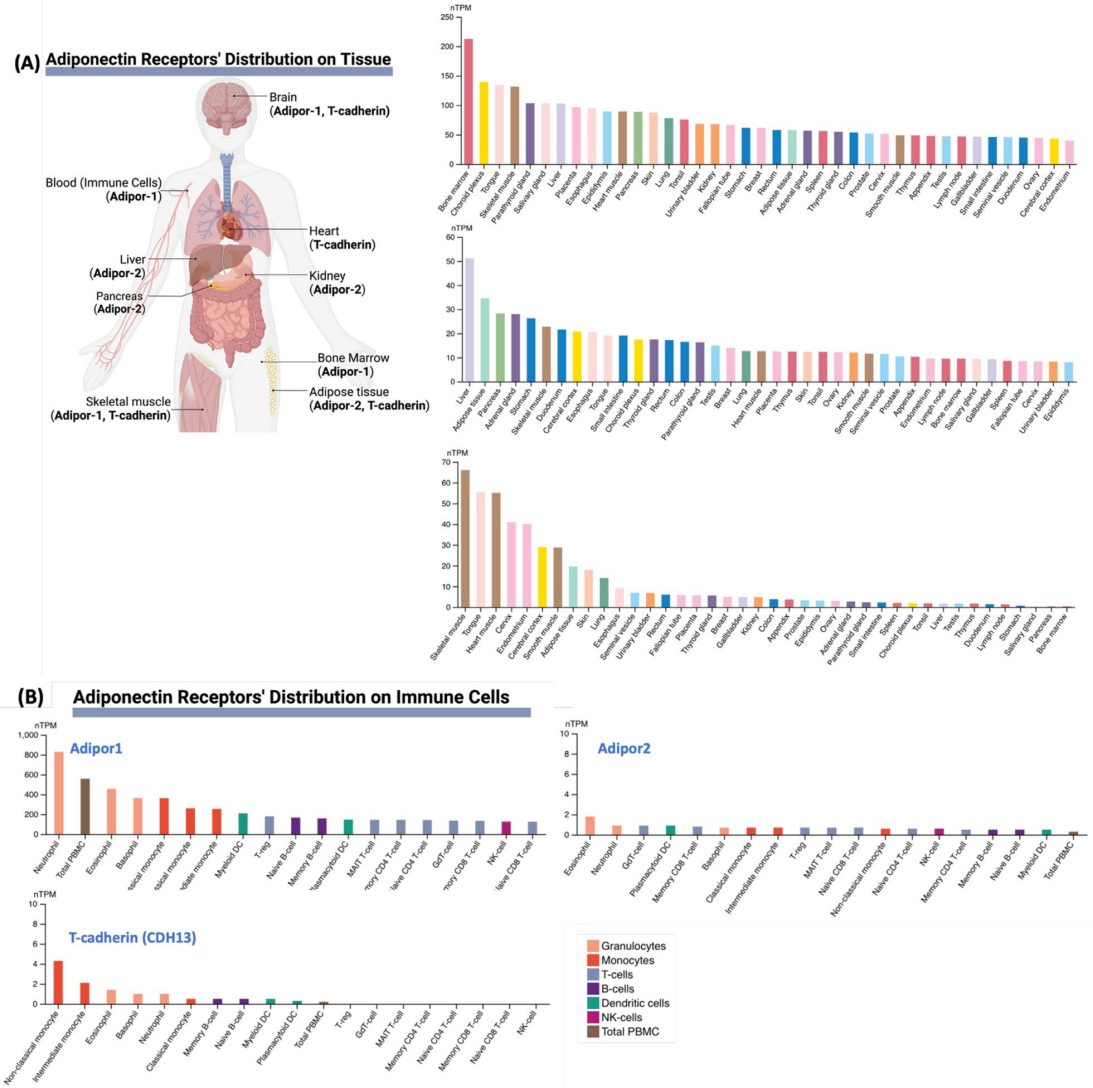
Adiponectin receptors distribution in (**A**) tissues and (**B**) immune cells. **(A)** AdipoR1 and T-cadherin are highly expressed in the brain and skeletal muscle. On the other hand, AdipoR2 is expressed in adipose tissue, liver, and pancreas. Interestingly, AdipoR1 is also highly expressed in bone marrow and **(B)** innate immune cells, especially granulocytes and macrophages. Data were extracted from the Human Protein Atlas (HPA) (https://www.proteinatlas.org). RNA expression was used for tissue distribution and single-cell RNA expression was used for immune cells

Collectively, these findings suggest that adiponectin receptor signaling plays a critical role in modulating both immune and metabolic responses during sepsis. The different functional roles (Fig. 2) along with the different expression and distribution profile of AdipoR1, AdipoR2, and T-cadherin (Fig. 3) highlight the importance of targeting specific adiponectin pathways to optimize therapeutic strategies. Future research should explore receptor-specific agonists or gene-modulating approaches to target adiponectin signaling in sepsis.

### Adiponectin and sepsis: clinical implications

#### The role of adiponectin in sepsis: clinical observations

Despite compelling findings from experimental models that suggest anti-inflammatory effects and tissue protection, clinical studies on adiponectin’s role in sepsis are limited, contradictory, and therefore and not that straightforward to interpret. While some clinical studies suggest that lower adiponectin levels correlate with worse sepsis prognosis as expected, others report that lower adiponectin is associated with better survival [99].

A study published by Hillenbrand et al. [100] investigated changes in adipokine levels among septic patients following major surgery. The researchers observed that median plasma adiponectin levels decreased after the onset of sepsis compared to pre-septic levels. Notably, patients who survived sepsis had significantly higher pre-septic adiponectin concentrations than those who did not survive. In survivors, adiponectin levels dropped by an average of 33% one day after sepsis onset, whereas non-survivors exhibited a slight increase of 11%.​ This study aligns with previous findings from the same group [101]. Another study by Venkatesh et al. [102] measured plasma adiponectin concentrations in critically ill patients on days 3 and 7 of their illness. The findings revealed that adiponectin levels were significantly lower in these patients compared to historical controls. Additionally, a strong correlation was identified between plasma adiponectin and cortisol levels on both days, suggesting a potential interaction between adiponectin and the stress response in critical illness.​ In addition, Wang et al.[103] reported that low plasma adiponectin levels at admission were associated with worse left ventricular cardiac function and higher 28-day mortality. Conversely, research published by Palakshappa et al. [104] examined the relationship between plasma adiponectin levels and the development of acute respiratory distress syndrome (ARDS) in patients with severe sepsis and septic shock. Contrary to expectations, the study found that higher adiponectin levels were associated with an increased risk of ARDS, and this association was independent of body mass index. Similarly, Koch et al.[105] and Walkey et al. [106] found that lower adiponectin levels at admission correlated with better sepsis survival.

Several factors may contribute to these discrepancies. For one, many clinical studies have small sample sizes and assess adiponectin levels only at a single time point rather than tracking changes throughout the disease course [107]. Since sepsis is a dynamic condition with shifting inflammatory states, the timing of adiponectin measurement seems to be critical to interpret these results. On this notion, longitudinal studies provide valuable insights into the role of adiponectin in sepsis progression. Vassiliadi et al. [26] examined septic patients requiring mechanical ventilation and found that adiponectin levels increased over time only in those who recovered. On the same note, Karampela et al. [107], Welters et al. [108] and Langouche et al. [109] suggested that rising adiponectin levels during sepsis may represent a compensatory response to excessive inflammation, facilitating immune resolution. Thus, higher adiponectin levels at admission may indicate excessive inflammation and could explain why they have been associated with lower survival in some cases. In line with the above, new insights on adiponectin’s role in disease suggest that its effects can be context-dependent, displaying both anti-inflammatory and pro-inflammatory roles based on disease states [2, 13].

In addition, sepsis is a complex, multifactorial condition influenced by a variety of factors that can affect its progression and clinical outcome. For instance, the concept of an “obesity paradox” in sepsis could further complicate the interpretation of adiponectin’s clinical implications in sepsis. Specifically, while obesity is a well-established risk factor for numerous chronic diseases, linked to chronic low-grade inflammation and impaired immune function, some studies suggest that higher body mass index (BMI) may confer a survival advantage in sepsis—a phenomenon known as the"obesity paradox"[110–115]. Given that obesity is typically associated with lower circulating adiponectin levels, this paradox adds complexity to the relationship between adiponectin and sepsis outcomes in patients.

Adding further complexity, adiponectin isoforms appear to have distinct roles in sepsis. High-molecular-weight (HMW) adiponectin, considered the most biologically active form, has been shown to increase in patients with clinical improvement while remaining suppressed in progressive sepsis [108]. Higher HMW levels correlate with reduced TNF-α, oxidative stress markers, and high-sensitivity C-reactive protein (hs-CRP), suggesting anti-inflammatory effects [116]. It also enhances glucose homeostasis and reduces inflammation via APPL1 (adaptor protein, phosphotyrosine interacting with PH domain and leucine zipper 1)-AMPK (AMP-activated protein kinase)-GLUT4 (glucose transporter 4) signaling in 3T3-L1 adipocytes under glucolipotoxic conditions [117]. Interestingly, HMW adiponectin can activate both AMPK, which exerts anti-inflammatory effects, and NF-κB, which is pro-inflammatory. Typically, AMPK activation dominates; however, transient NF-κB activation may initially promote inflammation before AMPK downregulates it in a dose-dependent manner. In metabolic diseases, a deficiency of HMW adiponectin may drive chronic inflammation by failing to suppress NF-κB, leading to oxidative stress and lipid accumulation [118]. Another important factor to consider when interpreting clinical studies is the variability of adiponectin levels within the general population. Women have higher levels of both total and HMW adiponectin vs men [119–124] and there is variation between ethnic groups. For example, Asians and Latinos have lower total and HMW adiponectin levels than Caucasians [125–127].

In most studies, the absolute level and HMW/total adiponectin ratio increase with progressing age [124, 128, 129]. MMW adiponectin is less potent than HMW but still plays a role in modulating inflammation and insulin sensitivity. While the research on MMW adiponectin in sepsis is more limited compared to HMW adiponectin, it is thought to contribute to some extent in regulating immune responses and metabolic functions. It may offer a protective effect in the early stages of sepsis by promoting insulin sensitivity and reducing systemic inflammation [130, 131]. Low-Molecular-Weight (LMW) Adiponectin, consisting primarily of trimers, is the least active isoform. It has limited effects on endothelial function and inflammation compared to the higher-molecular-weight forms but has been associated with metabolic disorders [132] and asthma [133]. In sepsis, LMW adiponectin may have a minimal impact on modulating the immune response and vascular integrity, though further research is necessary to fully understand its role.

Although adiponectin’s role in sepsis is influenced by various factors, including disease severity, patient heterogeneity, age, and sex, emerging evidence suggests that it holds therapeutic potential. Sepsis is a highly dynamic and multifactorial disease characterized by a dysregulated host immune response, leading in some cases, to chronic inflammation and organ dysfunction. Contrary to the earlier belief that sepsis progresses through distinct sequential phases—initial hyperinflammation followed by immunosuppression—recent evidence indicates that proinflammatory and anti-inflammatory responses can occur simultaneously from the early stages of the disease [22]. This concurrent activation contributes to the complexity of sepsis pathophysiology and has significant implications for therapeutic interventions. Given this complexity, treatment strategies must be adaptable to disease status and individual patient profiles. Despite its context-dependent effects, adiponectin exhibits immunomodulatory, metabolic, and endothelial-protective properties that could be leveraged for therapeutic benefit. Its fluctuating levels in response to sepsis severity suggest that it could serve as a valuable biomarker for patient stratification and risk assessment. While further research is needed to clarify its precise role, adiponectin’s potential in guiding personalized and precision-based treatments in sepsis should not be overlooked and is further discussed below. Table 2 provides a detailed summary of clinical studies investigating the role of adiponectin in sepsis, supporting either its anti-inflammatory or pro-inflammatory effects.

**Table 2:** Summary of clinical studies examining the role of adiponectin in sepsis. The table highlights findings that support both anti-inflammatory and pro-inflammatory effects

References	Adiponectin’s effect	Experimental approach	Key mechanism
Anti-inflammatory effects of adiponectin in clinical sepsis			
Hillenbrand et al., (2010) [100]	Hypoadiponectinemia predicts severity	Septic shock case–control	Sequential Organ Failure Assessment (SOFA)/Simplified Acute Physiology Score II (SAPS-II) correlations
Hillenbrand et al., (2016) [101]	High pre-septic levels predict better survival	Prospective surgical sepsis cohort	Metabolic resilience via AMPK
Venkatesh et al., (2009) [102]	Restoration marks clinical recovery	Longitudinal critical illness study	Inverse cortisol correlation (R^2^ = 0.64)
Wang et al., (2020) [103]	Cardioprotective in septic cardiomyopathy	Sepsis cohort with echocardiography	Preserved LVEF via AMPK
Compensatory/recovery effects of adiponectin in clinical sepsis			
Karampela et al., (2017) [107]	Convalescent increase reflects compensation	Sepsis-3 prospective cohort	Late-phase anti-inflammatory response
Welters et al., (2014) [108]	HMW rebound indicates recovery	Pilot sepsis serial sampling	HMW/total ratio normalization
Langouche et al., (2009) [109]	Normalization equals metabolic recovery	RCT sub-analysis (insulin therapy)	Insulin sensitivity correlation
Pro-inflammatory effects of adiponectin in clinical sepsis			
Palakshappa et al., (2016) [104]	Higher levels increase ARDS risk	Severe sepsis cohort	OR 1.12 per + 5 μg/mL (95%CI 1.01–1.25)
Koch et al., (2011) [105]	Elevated levels predict mortality	Mixed ICU prospective study	HR 1.67 for 3-year death
Walkey et al., (2010) [106]	Elevated levels associated with 28-day mortality	ARDS secondary analysis	OR 1.59 per + 5 μg/mL

### Potential adiponectin-based treatments for sepsis

Modulating adiponectin signaling or mimicking its effects has been proposed as a potential therapeutic strategy in sepsis. Among adipokines, adiponectin is secreted in the highest amounts, making it a particularly attractive target for intervention. Additionally, the potential role of GLP-1 receptor agonists (GLP-1RAs), which have been associated with increased adiponectin concentrations, presents an intriguing avenue for research in sepsis management. Below we discuss the potential of Adiponectin mimics and GLP-1RAs in treating sepsis based on current knowledge and pre-clinical studies and suggest possible clinical applications and future directions.

#### Reinforcing the adiponectin pathway: the case of AdipoR agonists

Several short peptides and small molecules have been identified today to target AdipoRs and mimic some of adiponectin's effects, primarily because direct use of adiponectin as a therapeutic agent has several challenges [134]. These agonists include AdipoRon, ALY688, Osmotin, ADP-1, ADP355, ADP399, Pep70, PEGylated BHD1028, GTDF and Tiliroside and can induce similar downstream signaling cascades to adiponectin, such as AMPK, p38MAPK, and PPARα [135]. While there is limited direct research on AdipoR agonists in sepsis, AdipoRon has shown promise in metabolic and inflammatory disorders [136], suggesting potential applicability in sepsis. Specifically, literature supports various effects of AdipoRon through its action on adiponectin receptors (AdipoR1 and AdipoR2), that could be beneficial for sepsis:

- *Anti-Inflammatory Effects* Sepsis is characterized by a systemic hyper-inflammatory response, often leading to tissue injury and organ dysfunction. AdipoRon has demonstrated the ability to downregulate the production of pro-inflammatory cytokines such as TNF-α, IL-1β, and IL-6 while enhancing anti-inflammatory cytokines like IL-10 both in systemic [137] and tissue inflammation [138], helping to restore immune balance and mitigate the damaging effects of an uncontrolled inflammatory response.
- *Improvement of Endothelial Function* Endothelial dysfunction is a critical aspect of sepsis, contributing to increased vascular permeability and the development of septic shock. AdipoRon enhances endothelial function by promoting nitric oxide (NO) production and reducing oxidative stress [139]. This action helps maintain vascular integrity and prevent complications associated with sepsis, such as acute respiratory distress syndrome (ARDS).
- *Metabolic Regulation* Sepsis often leads to metabolic dysregulation, including insulin resistance and dyslipidemia. AdipoRon has been shown to improve insulin sensitivity and lipid metabolism, which can help stabilize blood glucose levels and restore metabolic homeostasis in septic patients [140, 141]. By promoting better metabolic parameters, AdipoRon can mitigate the negative effects of sepsis on organ function.
- *Oxidative Stress Reduction* Elevated oxidative stress is common in septic conditions and can contribute to cellular injury. AdipoRon exhibits antioxidant properties, helping to scavenge reactive oxygen species (ROS) and reduce oxidative damage [142, 143]. This protective effect can further enhance cellular resilience during sepsis.

More importantly, very recent studies have shown that AdipoRon alleviates memory impairment in sepsis [56] and endotoxin-induced acute hepatitis [144] in mice, further supporting adiponectin’s potential as a therapeutic agent for sepsis. Despite these encouraging findings, Adipor-agonists, including AdipoRon, remain in the preclinical development stage and are not approved for clinical use. Nevertheless, their immunomodulatory and anti-inflammatory properties support their potential as a novel therapeutic approach in sepsis along with other critical illnesses, highlighting the need for further pharmacological optimization and eventual clinical evaluation [145].

#### Reinforcing the adiponectin pathway: the case of GLP-1 receptor agonists

Glucagon-like peptide-1 receptor agonists (GLP-1RAs) are primarily used for treating obesity and managing type 2 diabetes. Notable GLP-1RAs include Exenatide, Lixisenatide, Liraglutide, Dulaglutide, and Semaglutide. These medications mimic glucagon-like peptide-1 (GLP-1), a hormone released after food intake, to enhance insulin secretion, inhibit glucagon release, and slow gastric emptying, thereby regulating blood sugar levels [146]. Given the established link between obesity and sepsis outcomes, GLP-1RAs have gained interest as potential therapeutic agents for sepsis.

Beyond their role in glycemic control, GLP-1RAs interact with various immune cells, exerting anti-inflammatory and organ-protective effects. This immunomodulatory capacity makes them promising candidates for managing sepsis, particularly due to their ability to mitigate inflammation and organ dysfunction [147]. Emerging evidence further supports this hypothesis, as GLP-1RAs have been shown to influence adiponectin levels positively [36] potentially improving sepsis outcomes by addressing both metabolic regulation and inflammation [148, 149]. Mechanistically, GLP-1 activates cAMP signaling in adipocytes, leading to increased adiponectin secretion via protein kinase A (PKA) activation [150], assuming that GLP-1RAs act through the same pathway. In support, Exendin-4, a peptide derived from the Gila monster lizard venom, a naturally occurring GLP-1 receptor agonist, has also been demonstrated to reduce inflammatory responses in macrophages by upregulating adiponectin levels [151]. This link between GLP-1RAs and adiponectin suggests a mechanism for reducing hyperinflammation, minimizing tissue injury, and improving survival rates in sepsis. By increasing adiponectin levels, GLP-1RAs may also alleviate endothelial dysfunction and oxidative stress, two critical factors in sepsis pathology. While human data remain limited, preclinical studies using murine models of sepsis highlight the beneficial effects of GLP-1RAs, including reduced pro-inflammatory cytokine production and decreased organ injury. For example, Yang et al. [149], found that GLP-1RAs inhibit NF-κB activation and oxidative stress, ultimately enhancing survival in sepsis models. Similarly, Sato et al. [152] demonstrated that liraglutide treatment improved outcomes in experimental models of lung injury. Additionally, GLP-1RAs may provide therapeutic benefits in sepsis-induced acute kidney injury (AKI) through metabolic reprogramming, as research indicates that early metabolic alterations during sepsis significantly impact organ dysfunction and the progression to chronic kidney disease [153]. Supporting this, studies have shown that GLP-1 receptor expression in renal tubules increases during early sepsis, suggesting a role for endogenous GLP-1 in counteracting sepsis-related inflammation [154]. The combined metabolic and immune-modulating effects of GLP-1RAs, potentially in conjunction with adiponectin, highlight their promise in sepsis management.

#### Reinforcing the adiponectin pathway: clinical observations

Specific adiponectin-targeted therapies have limited evaluation in clinical studies so far. Some proof-of-concept comes from initial clinical observations on GLP-1RA use, suggest that it may lower the risk of severe infections and organ dysfunction compared to other anti-diabetic medications. A study by Alex E. Henney et al. [155] indicated that GLP-1RAs are linked to reduced incidences of pneumonia and severe sepsis in type 2 diabetes patients. Briefly, this retrospective cohort study evaluated whether GLP-1RAs reduce the risk of pneumonia and severe sepsis. compared to dipeptidyl peptidase-4 inhibitors (DPP-4i). In 331,863 matched GLP-1 RA users, pneumonia (HR 0.60, 95% CI 0.58–0.62) and severe sepsis (HR 0.61, 95% CI 0.59–0.63) risks were significantly reduced compared to the DPP-4i arm. In another very recent preliminary study, it was shown that patients on GLP-1RAs prior to hospitalization had a lower risk of sepsis and organ failure compared to those on other anti-diabetic agents, supporting the potential protective role of GLP-1RAs in sepsis. Specifically, the study conducted a retrospective cohort analysis to evaluate whether pre-hospital GLP-1RA exposure reduces sepsis and organ failure risk in adults hospitalized with infection. Patients on GLP-1RAs were compared to those receiving Dipeptidyl Peptidase-4 inhibitors (DPP-4is) and Sodium-Glucose Cotransporter-2 inhibitors (SGLT2is). Among 353 GLP-1RA users, 860 DPP-4i users, and 152 SGLT2i users, the median patient age was 62, and most had diabetes (95.8%) or obesity (62.1%). Overall, 32.3% developed sepsis. Compared to DPP-4i users, GLP-1RA users had significantly lower odds of developing sepsis (OR = 0.69, p = 0.010), hepatic failure (OR = 0.42, p = 0.031), and any organ failure (OR = 0.66, p = 0.008). Similar trends were observed when comparing GLP-1RA users to SGLT2i users, though statistical significance was limited by the smaller sample size [156]. Future clinical trials are necessary to establish a causal relationship between elevated adiponectin levels and improved sepsis outcomes, paving the way for targeted therapeutic strategies.

## Future directions and research priorities

While the current evidence highlights adiponectin as a significant modulator in sepsis pathophysiology, substantial research gaps remain before its therapeutic potential can be fully realized in clinical practice. Addressing these gaps requires a multi-pronged approach encompassing fundamental mechanistic studies, robust clinical trials, diagnostic advancements, and the development of targeted therapeutic strategies.

A critical imperative is the execution of well-designed, longitudinal clinical trials tracking the dynamics of specific adiponectin isoforms (HMW, MMW, LMW) throughout the course of sepsis. These studies must enroll diverse patient populations, considering variations in age, sex, ethnicity, underlying comorbidities (including obesity status), and sepsis etiology and severity. Such dynamic tracking is essential to resolve the conflicting findings in current clinical literature regarding adiponectin's prognostic value and to understand how isoform profiles correlate with disease progression, inflammatory states (hyperinflammation vs. immunosuppression), organ dysfunction, and ultimate outcomes. Concurrently, further mechanistic research is crucial to definitively clarify the context-dependent roles of adiponectin. Studies must elucidate the precise molecular pathways determining whether adiponectin exerts pro- or anti-inflammatory effects at different stages of sepsis and within specific organ microenvironments. This includes investigating downstream signaling via AdipoR1, AdipoR2, and T-cadherin, interactions with other inflammatory mediators (like HMGB1, TNF-α, IL-6, IL-10), and its influence on specific immune cell functions (macrophage polarization, neutrophil activity, T cell responses) during the evolving septic state. Addressing the"obesity paradox"in sepsis represents a specific challenge within this mechanistic framework; dedicated research is needed to understand adiponectin's contribution (or lack thereof) to the observed survival differences in obese versus non-obese septic patients, potentially linking it to baseline inflammatory status, adipose tissue dysfunction, and differential isoform secretion.

Complementary to therapeutic development, advancing diagnostic capabilities is paramount. There is a pressing need to develop and validate rapid, reliable, and potentially point-of-care assays capable of quantifying not just total adiponectin but, critically, its distinct isoforms (especially HMW). Such assays would enable clinicians to potentially use adiponectin isoform profiles for risk stratification, real-time monitoring of disease progression, and guiding therapeutic decisions, moving towards more personalized sepsis care.

On the therapeutic front, direct modulation of the adiponectin pathway holds promise. Rigorous preclinical studies are required for direct head-to-head comparisons and detailed dose-finding evaluations of AdipoR agonists, such as AdipoRon, using clinically relevant animal models of sepsis that replicate key features of human disease (e.g., varying pathogens, comorbidities). These studies should assess efficacy in improving survival, reducing organ injury (particularly relevant for SAE, AKI, ARDS), and modulating inflammatory responses. Success in preclinical models would pave the way for carefully designed Phase I/II human clinical trials to assess safety, tolerability, pharmacokinetics, pharmacodynamics (including impact on adiponectin isoform levels and downstream biomarkers), and preliminary efficacy of AdipoR agonists in septic patients.

Furthermore, the potential repurposing of GLP-1 Receptor Agonists (GLP-1RAs) warrants specific investigation in the context of sepsis. While promising preliminary data exist, large-scale, prospective clinical trials are needed to specifically evaluate GLP-1RAs for sepsis-related outcomes, beyond their established use in diabetes and obesity. These trials must include non-diabetic septic patients to isolate the effects independent of glycemic control and should rigorously assess the impact of GLP-1RA administration on both clinical endpoints (e.g., mortality, organ failure scores, duration of ventilation/ICU stay) and circulating adiponectin levels (total and isoforms) to confirm the mechanistic link.

Recognizing the complexity of sepsis pathophysiology, investigation into combination therapies is essential. Future studies should explore the potential synergistic benefits of combining adiponectin pathway modulators (e.g., AdipoR agonists, GLP-1RAs) with components of standard sepsis care, such as specific antimicrobial strategies, immunomodulatory agents currently under investigation, or targeted organ support techniques. The goal is to identify regimens that address multiple facets of the dysregulated host response simultaneously.

Ultimately, the successful translation of adiponectin-targeted strategies into clinical practice will depend on adopting a precision medicine approach. Future research must focus on defining patient subgroups most likely to benefit from these interventions. This requires integrating data from dynamic biomarker monitoring (including adiponectin isoforms), clinical characteristics (obesity status, baseline metabolic health), genetic factors, inflammatory phenotypes (e.g., based on cytokine profiles), and the specific stage or trajectory of sepsis. Identifying predictive biomarkers and patient signatures will be pivotal for tailoring adiponectin-based therapies effectively.

## Conclusion

In conclusion, adiponectin emerges as a critical player in the pathophysiology of sepsis, linking metabolic and inflammatory responses in a complex interplay that can significantly influence patient outcomes. Despite the existing “obesity paradox” complicating the understanding of adiponectin's role in sepsis, emerging evidence suggests that its anti-inflammatory properties may provide protective effects during septic events. The potential of enhancing adiponectin signaling through agents like AdipoRon could offer a promising therapeutic strategy to improve immune modulation, reduce organ dysfunction, and promote recovery in septic patients. Furthermore, GLP-1 receptor agonists (GLP-1RAs) represent an exciting avenue for sepsis management, not only through their metabolic benefits in obesity and type 2 diabetes but also by their ability to elevate adiponectin levels. This dual action may help mitigate hyperinflammation and tissue injury associated with sepsis. As research progresses, identifying additional compounds and interventions that modulate adiponectin pathways could open new therapeutic avenues, offering innovative strategies to harness its protective effects against inflammation and organ dysfunction in sepsis. By focusing on the development of targeted therapies that boost adiponectin activity, we may significantly improve patient outcomes and pave the way for more effective interventions in critical care settings. Future clinical trials will be essential to confirm these benefits and refine approaches tailored to individual patient profiles. In addition, the signaling pathways associated with adiponectin show nuanced effects depending on the inflammatory context. Future research should explore the context-specific roles of adiponectin and AdipoR-and GLP1 RAs in sepsis management to optimize therapeutic decisions. Understanding how these molecules interact with different immune responses will be vital for ensuring that patients receive the most appropriate and effective treatments.

## Data Availability

No datasets were generated or analysed during the current study.
